# Proton-Shuttling Lichen Compound Usnic Acid Affects Mitochondrial and Lysosomal Function in Cancer Cells

**DOI:** 10.1371/journal.pone.0051296

**Published:** 2012-12-05

**Authors:** Margret Bessadottir, Mar Egilsson, Eydis Einarsdottir, Iris H. Magnusdottir, Margret H. Ogmundsdottir, Sesselja Omarsdottir, Helga M. Ogmundsdottir

**Affiliations:** 1 Faculty of Medicine, University of Iceland, Reykjavik, Iceland; 2 Faculty of Pharmaceutical Sciences, University of Iceland, Reykjavik, Iceland; Technische Universitaet Muenchen, Germany

## Abstract

The lichen compound usnic acid (UA) is a lipophilic weak acid that acts as a proton shuttle and causes loss of mitochondrial inner membrane potential. In the current study we show that UA treatment induced the formation of autophagosomes in human cancer cells, but had minimal effects on normal human fibroblasts. However, autophagic flux was incomplete, degradation of autophagosomal content did not occur and acidification was defective. UA-treated cells showed reduced ATP levels and activation of AMP kinase as well as signs of cellular stress. UA is thus likely to trigger autophagosome formation both by energy depletion and stress conditions. Our findings indicate that the H^+^-shuttling effect of UA operates not only in mitochondria as previously shown, but also in lysosomes, and have implications for therapeutic manipulation of autophagy and pH-determined drug distribution.

## Introduction

Lichens, the symbiosis between a fungal partner and a photobiotic microorganism, are found all around the world and give rise to a large number of unique secondary metabolites [1]. The dibenzofuran derivative, usnic acid (UA) is a known secondary metabolite and has been studied to some extent [2]. A wide range of biological activities has been reported for usnic acid, e.g. anti-microbial, anti-viral, anti-pyretic, anti-inflammatory and analgesic effects [2]. Anti-tumor activity of UA was first reported three decades ago in lung carcinoma in mice and in P388 leukemia [3], [4]. It has furthermore been shown that usnic acid has anti-mitotic effects on human cancer cell lines [5] and causes a loss of viable cells in leukemia, lung and breast cancer cells [6], [7]. However, exposure to UA does not activate p53 and has not been proposed to be involved in DNA damage [8].

UA is a lipophilic weak acid (p*K*a 4.4) that can cause proton leakage by diffusing through mitochondrial membranes [9]. In mouse liver cells usnic acid disrupts the normal metabolic processes of cells by uncoupling oxidative phosphorylation in mitochondria and by activating oxidative stress [10]. Mitochondria play an important role in the regulation of cell death pathways and mitochondrial changes have been described in cancer cells, including increased stability, thus inhibiting the release of cytochrome *c* and preventing induction of apoptosis [11]. Our previous study showed that UA treatment causes loss of mitochondrial membrane potential in two different cancer cell lines [12]. Interestingly, it has been shown that changes in mitochondrial membrane potential can lead to the onset of autophagy [13].

Autophagy is a process that can both aid cancer cell survival during nutrient shortage but can also promote cancer cell death. The molecular pathways that determine this dual role remain obscure and it is likely that the function of autophagy in cancer depends on tumor stage, cellular context and tissue of origin [14], [15]. More than 30 different protein encoding genes, known as autophagy-related genes (ATG), have been identified and studies in mouse models have shown that macroautophagy is essential for maintenance of cellular homeostasis in many tissues [16], [17]. Autophagy can be triggered by nutrition depletion or metabolic stress and can vary depending on the demand for substrate degradation and stimulus. The energy sensor AMP kinase signals to the mammalian target of rapamycin complex 1 (mTORC1), a major regulator of autophagy, which directly controls protein synthesis and anabolic processes in a nutrient-sensitive manner. Starvation-induced autophagic vesicles are formed, which are likely to contain free cytosol [18], [19]. Additionally, other stress conditions such as damaged organelles, intracellular pathogens or stress in the endoplasmic reticulum can induce autophagy through different pathways from those activated by starvation [19]. The maturation process, the final step of autophagy, involves delivery and degradation of autophagic cargo. Fusion occurs with lysosomes, and autophagic vesicles coalesce and contents are degraded. The acidic environment of lysosomes is essential for the final steps of autophagy, and by disrupting the vacuolar H^+^ ATPase, which is involved in acidifying lysosomes, the completion of autophagy can be inhibited [15], [19].

The aim of present study was to explore further the consequences of loss of mitochondrial membrane potential induced by UA. We asked whether this caused release of cytochrome *c* and triggered apoptosis. Loss of membrane potential and the property of UA to shuttle protons across membranes would be expected to lead to a decline in ATP production by mitochondria [9]. We found that UA treated cancer cells had decreased ATP levels and increased phosphorylation of AMP kinase. Interestingly, UA triggered autophagy but without degradation of autophagosomal content, suggesting a disruptive effect on autophagolysosomal acidification. Our results indicate that the induction of autophagy was mediated by a combination of response to nutrient shortage as well as cellular stress.

**Figure 1:**
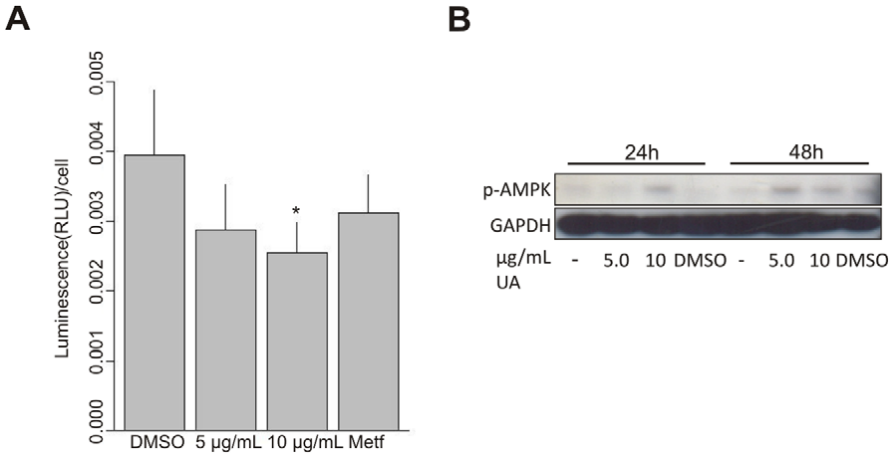
UA caused decline in cellular ATP and activation of AMP kinase. (A) Levels of ATP, measured in a luminometer, were decreased in T47D cells after treatment with UA (10 µg/mL; DMSO 0.2%) for 24 hours. Data are presented as luminescence/cell of each group compared with DMSO control. Error bars indicate standard error of the mean, *p<0.05. (B) Phosphorylation of AMP kinase, verified by Western blotting, was detected in T47D cells after treatment with UA (10 µg/mL; DMSO 0.2%) for 24 and 48 hours.

## Materials and Methods

### Plant Material, Cell Culture and Exposure to Test Substances

(+)-Usnic acid (97%) was isolated from *Cladonia arbuscula* (Wallr.) Rabenh. (Cladoniaceae) collected in open country in Iceland, not privately owned. Isolation and identification was performed as described [12]. The substance was dissolved in dimethyl sulfoxide (DMSO; Merck, 2951) and diluted for use in tissue-culture medium. All tests included controls where the highest equivalent concentration of DMSO was used. The breast cancer cell lines T47D and MCF7 and the pancreatic cancer cell line Capan-2 were obtained from the American Type Culture Collection (ATTC) through LGC Promochem. T47D contains a single mutated copy of p53 [20], but Capan-2 and MCF7 are homozygous for wild-type p53 [21]. MCF7 is estrogen receptor positive [22]. Primary human fibroblasts were cultured from normal skin biopsies and used in passage 6–13 (National Bioethics Committee permission VSNb2006020001/03-16; informed consent obtained). All cell lines were maintained in RPMI-1640 tissue culture medium (GIBCO™, 52400), containing 0.5% penicillin and streptomycin (GIBCO™, 15140-148) and 10% heat-inactivated fetal bovine serum (FBS; GIBCO™, 10270) with T47D receiving additionally 0.01 mg/mL insulin (Sigma, I1882) and subcultured following detachment by trypsin (0.25% Trypsin/EDTA, Difco™, 215240) as appropriate. Cells were seeded at an appropriate number to exceed 70–80% confluence after 24 hour culture. (+)-Usnic acid (5 or 10 µg/mL), metformin (10 mM; Sigma, D150959) and solvent control were added and the cells were incubated under standard conditions, for different time periods. For the induction of autophagy by nutrient deprivation, cells were incubated with Hank’s solution (Sigma, H9394) for the last 40 min of the incubation time.

**Figure 2:**
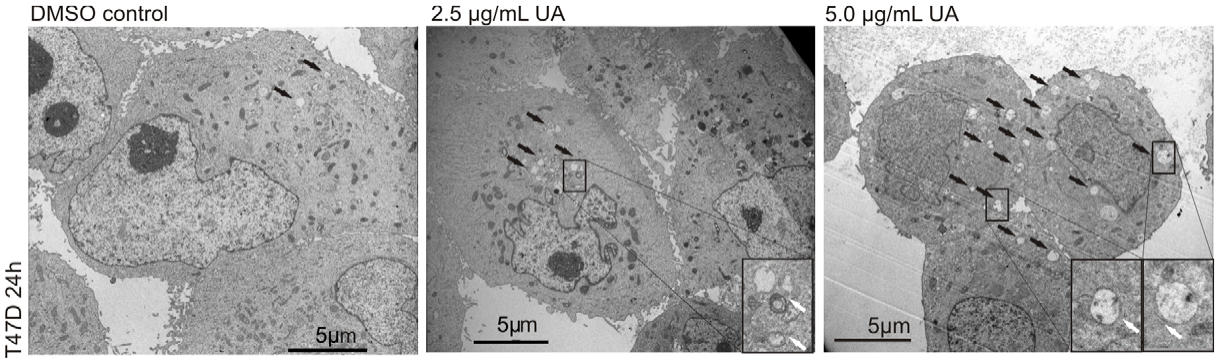
UA induced formation of autophagosome vacuoles. Induction of autophagic vacuoles, with double membranes characteristic of autophagosomes, was detected by electron microscopy in T47D cells after treatment with UA (2.5 and 5.0 µg/mL; DMSO 0.1%) for 24 hours. Black arrows indicate autophagic vacuoles, white arrows indicate double membrane formation.

**Figure 3:**
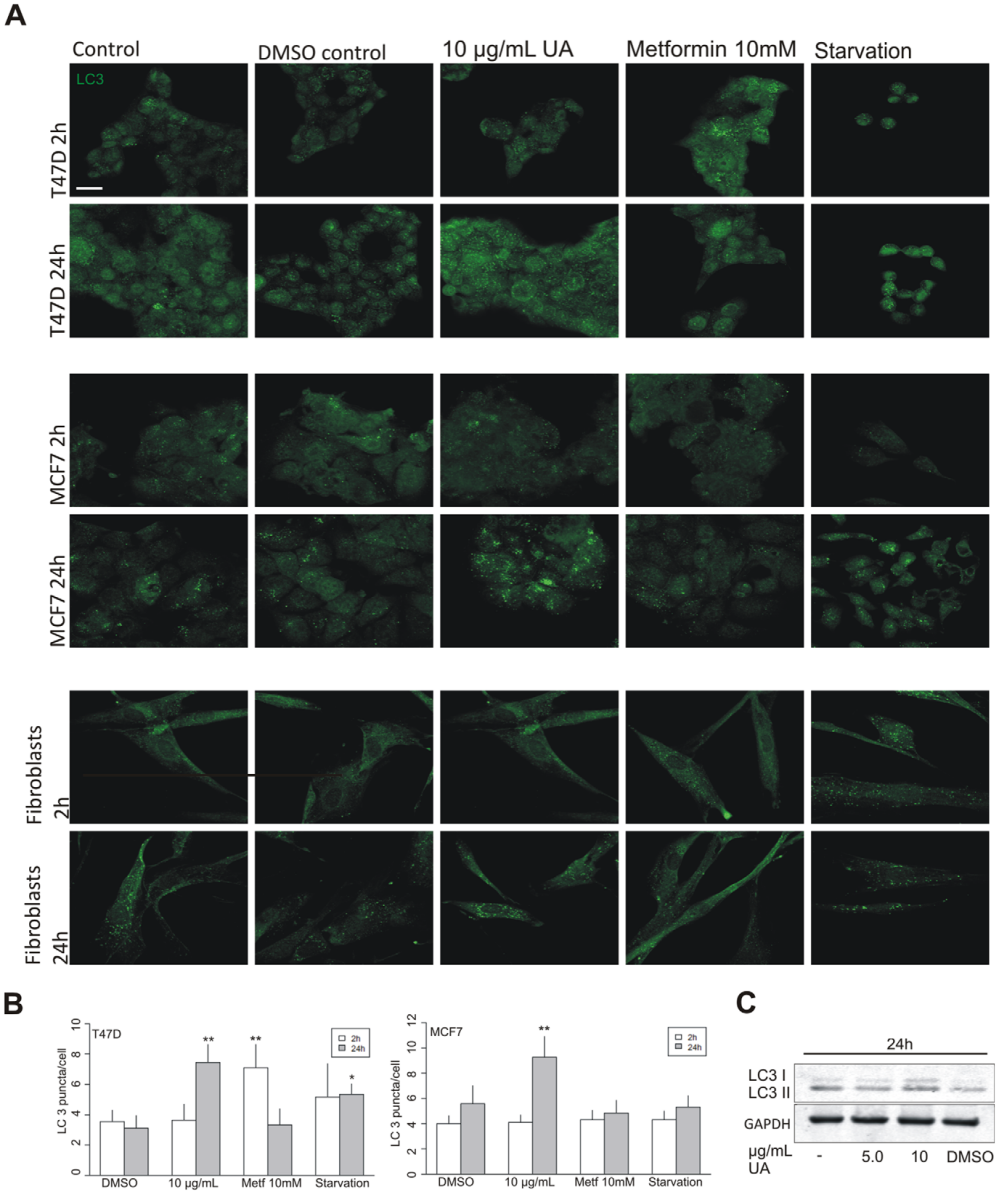
UA induced formation of LC3 puncta. (A) An increase in LC3 puncta was detected, by immunofluorescence in T47D and MCF7 cells after treatment with UA (10 µg/mL) for 24 hours. No effect was seen in normal fibroblasts. The scale bar shown represents 20 µm and applies to all panels. (B) LC3 puncta per cell were counted and quantified by ImageJ and data represented as LC3 puncta/cell of each group compared with DMSO control. Error bars indicate standard error of the mean, *p<0.05, **p<0.001. (C) Increase in LC3 I and LC3 II, verified by Western blotting, was detected in T47D cells after treatment with UA (10 µg/mL; DMSO 0.2%) for 24 hours.

### Estimation of Levels of ATP

Cells were detached by trypsinization, a small aliquot removed for counting and harvested using 0.5 M perchloric acid (Merck, 1.00519) for 10 min at 4°C. After centrifugation 10 µL of the supernatant were mixed with 1 mL of distilled water. Bioluminescence was assayed using 75 µL luciferase reagent (Promega, FF2021) which lysed the cells and provided the substrate luciferin. Luminescence was measured in a luminometer (Turner TD 20/20) and expressed as luminescence/cell.

**Figure 4:**
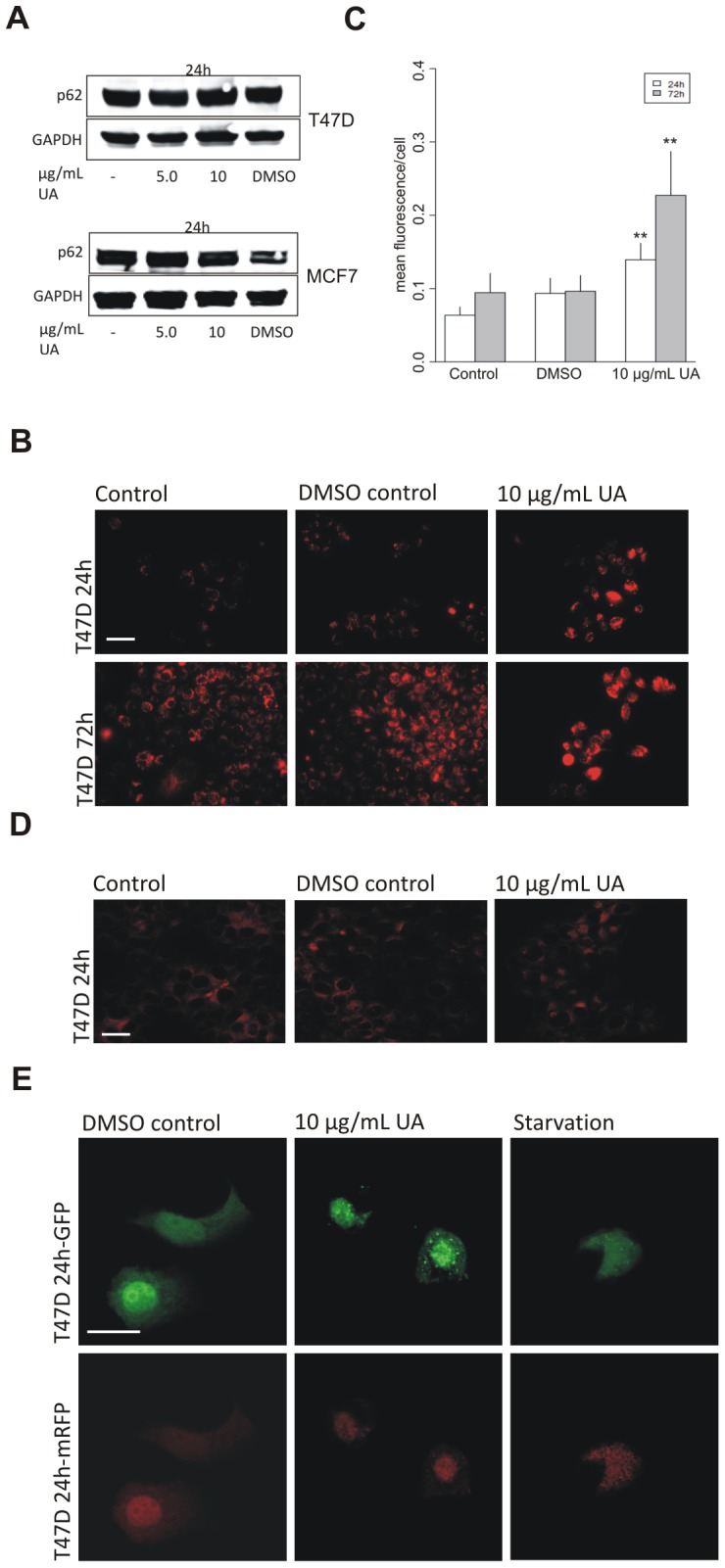
UA-induced formation of autophagosomal vaculoes was not followed by autosomal maturation and substrate degradation. (A) No degradation of p62 was detected, by Western blotting, in T47D and MCF7 cells after treatment with UA (5.0 and 10 µg/mL, 24 h; DMSO 0.1%). (B) Lysotracker, detected by fluorescence microscopy, shows diffuse staining in T47D cells after treatment with UA (10 µg/mL; DMSO 0.2%) for 24 h and 72 h. (C) Lysotracker intensity per cell was quantified by ImageJ and data presented as mean fluorescence value of each group compared to DMSO control. Error bars indicate standard error of the mean, **p<0.001. The scale bar shown represents 100 µm and applies to all panels. (D) No effects on Lamp2 immunostaining were detected, after treatment with UA *(*10 µg/mL; DMSO 0.2%) for 24 hours. The scale bar shown represents 100 µm and applies to all panels. (E) A plasmid expressing mRFP-GFP-LC3 was transfected into T47D cells. Lack of autophagolysosomal acidification was seen after treatment with UA (10 µg/mL; DMSO 0.2%) for 24 hours by detection of distinct GFP puncta. The scale bar shown represents 20 µm and applies to all panels.

### Electron Microscopy

After 24 hours’ incubation time at standard conditions cells were harvested by trypsinization and fixed in 1 mL of glutaraldehyde (Ted Pella Inc, 18426) solution for 60 min at room temperature, then centrifuged and stored at 0–5°C for 24 hours. After removing the glutaraldehyde, two drops of a 2% gelatinized solution (Ted Pella Inc, 19225) of distilled water were added to the cell pellet, carefully mixed and stored at 0–5°C for 24 hours. The samples were then washed twice with PBS and osmium tetroxide (Ted Pella Inc, 18463) added to each sample for one hour and washed again with PBS. Samples were cut to 2–5 mm pieces under a macroscope using razor blades. The pieces were dehydrated using ethanol (Merck, 64271D) at increasing concentrations, under rotation. Epoxy-resin (Ted Pella Inc, 18300) was added, first at 1∶1 (vol:vol) with 99% ethanol (Merck, 64271) for one hour, then twice resin only for one hour each time. The moulds were then placed in an oven at 70°C for 24 hours. The resin was then sliced with a glass knife (thickness about 0.5 µm) and stained with toluidine blue for selection of samples for sectioning with a diamond knife (70–100 nm thickness). The samples were placed on a copper frame before staining with a 0.06 g/mL lead-citrate solution (Ted Pella Inc, 19314) and were visualized using a Philips EM300 electron microscope. Images projected were developed using standard procedures for photographing. The developed film was scanned into a computer with a Nikon Coolscan V ED.

**Figure 5:**
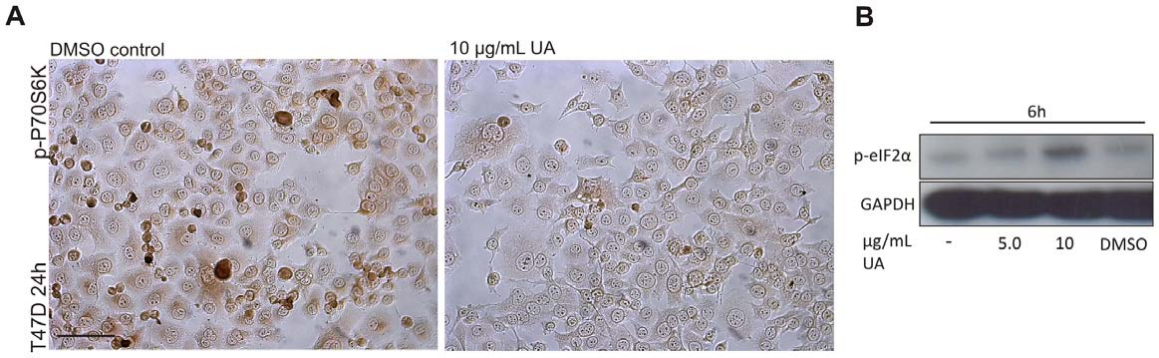
Formation of autophagosomes following UA treatment is likely to be induced through two different pathways. (A) A decrease in p-P70S6K was detected, by immunoperoxidase staining, after treatment with UA (10 µg/mL; DMSO 0.2%) for 24 hours. The scale bar shown represents 100 µm and applies to both panels. (B) An increase in p-eIF2α was detected, after treatment with UA (10 µg/mL; DMSO 0.2%) for 6 hours.

### Immunocytochemistry

For immunofluorescence staining, cells were harvested and fixed in 4% paraformaldehyde (Sigma, P6148) and stained with anti-cytochrome *c,* mouse IgG2a monoclonal antibody (Abcam, ab110325), cleaved caspase-3, rabbit polyclonal antibody (Cell Signaling, 9661) or LAMP2, mouse IgG1 monoclonal antibody (H4b4, obtained from University School of Medicine, Baltimore), followed by Alexa Fluor red 546 goat anti-rabbit IgG antibody (Invitrogen, A11010), Alexa Fluor green 488 goat anti-rabbit IgG antibody (Invitrogen, A11070) or Alexa fluor red 546 goat anti-mouse IgG_2a_ antibody (Molecular Probes, A11018) For nuclear staining TO-PRO-3 iodide (Invitrogen, T3605) was used. For the LC3 detection the cells were fixed with methanol (Sigma, 34860) for 10 min at −20°C and stained with anti-LC3B (D11), rabbit IgG monoclonal antibody (Sigma, L7543) followed by Alexa Fluor green 488 goat anti-rabbit IgG antibody (Invitrogen, A11070). The stained cells were visualized and photographed under a confocal microscope (Zeiss, LSM 5 Pascal). For the immunoperoxidase staining cells were fixed with methanol (Sigma, 34860) for 5 min at −20°C and stained with anti-phospho-p70S6 kinase (Thr389; 108D2), rabbit IgG, monoclonal antibody (Cell Signaling, 9234), anti-LC3B (D11), rabbit IgG monoclonal antibody (Sigma, L7543) and anti-p62 (SQSTM1), rabbit polyclonal antibody (Enzo, PW9860) followed by incubation with monoclonal mouse anti-rabbit immunoglobulins IgG1κ (Dako, M0737), polyclonal rabbit anti-mouse immunoglobulins IgG (Dako, Z0259), PAP, horseradish peroxidase and mouse monoclonal anti-horseradish immunocomplexes, IgG1 (Dako, P850) and DAB tablets, chromogen (Dako, S3000). The stained cells were visualized and photographed under a under light microscope (Leica DMI 3000B).

### Western Blot Analysis

Cells were harvested and lysed with RIPA buffer. Protein content was quantified spectrometrically using Bradford reagent (Sigma, B6916). Proteins were separated on NuPAGE 10% Bis-Tris Mini Gels and transferred to 0.2 µM polyvinylidene difluoride (PVDF) membrane by electroblotting. Membranes were probed with anti-phospho-AMPKα (Thr172) rabbit IgG monoclonal antibodies (Cell Signaling, 4188), anti-p62 (SQSTM1), rabbit polyclonal antibody (Enzo, PW9860), anti-LC3B (D11), rabbit IgG monoclonal antibody (Sigma, L7543), anti-phospho-eIF2α (Ser51) rabbit polyclonal antibody (Cell Signaling, 9721) or anti-G3PDH rabbit anti-human polyclonal antibody (R&D Systems, 2275-PC-1). Secondary antibody used was goat anti-rabbit IgG/HRPlinked (Cell Signaling, 7074S) and secondary antibody conjugated to IRDye-680 or 800 (Metabion, 68021). Proteins were visualized by the enhanced chemiluminescence (ECL) detection kit (GE Healthcare, RPN2132) and the signal was detected using a high performance chemiluminescence film (GE Healthcare, 91415) or detected by Odyssey infrared imaging system.

### Visualization of Lysosomes by LysoTracker Probes

The tissue culture medium was replaced by pre-warmed (37°C) 75 nM Lysotracker Red DND-99 (Invitrogen, L7528) and cells incubated at 37°C for 1 h. Loading solution was then washed of and replaced by fresh medium and the stained cells were visualized and photographed under fluorescence microscope (Leica DMI 3000B). Lysotracker is a fluorescent acidotropic probe for labeling and tracing acidic organelles in cells. The protonated form of this probe accumulates in acidic compartments, where it forms aggregates that fluoresce bright red.

### Transfection with tfLC3 Construct

The plasmid mRFP-GFP tandem fluorescent-tagged LC3 (tfLC3) construct was kindly provided by Prof. Kevin Ryan, Beatson Institute, Univeristy of Glasgow, with permission from Prof. Tamotsu Yoshimori, Osaka University [23], [24]. Calcium/manganese based (CCMB) transformation of DH10B strains of E.coli was used as previously described [25]. Transfection was performed using TransPass D2 (BioLabs, M2554S) according to the manufacturer’s protocol. After transfection cells were exposed to test substances or deprived of nutrients as described above. Cells were harvested and fixed in 4% paraformaldehyde (Sigma, P6148), and visualized and photographed under a confocal microscope (Zeiss, LSM 5 Pascal).

### Statistical Analyses

Statistical comparisons of mean values were performed using two sided analysis of variance (ANOVA), including the treatment and number of run as factors, followed by a post. hoc comparison using Tukey HSD. p values are described in the text at appropriate points. On all figures, * and ** indicate p<0.05 and p<0.001 respectively. Images and data shown are representative of what was observed in at least three separate experiments.

## Results and Discussion

The intrinsic pathway of apoptosis is triggered by opening of pores into the outer mitochondrial membrane leading to release of cytochrome *c* into the cytosol and activation of the caspase cascade [26]. To follow up on our previous work on the effects of UA on mitochondrial membrane potential [12] we investigated cytochrome *c* leakage and cleavage of caspase-3 by immunostaining in the breast cancer cell line T47D and the pancreatic cell line Capan-2. No cytochrome *c* release or cleaved caspase-3 products were detectable after treatment with usnic acid (10 µg/mL) after 24, 48 and 72 hours (data shown for 72 hours; Fig. S1A and Fig. S1B). These results support our previous data that UA causes late necrosis but no apoptosis [12], and indicate that, although the mitochondrial pH gradient is disrupted, the mitochondria themselves are intact.

It has been reported that usnic acid causes uncoupling of mitochondria [27], inhibits mitochondrial respiration and causes a drop in ATP levels in murine hepatocytes [10]. Gene expression data from microarray analysis has strengthened the suggestion that usnic acid shuttles protons against the gradient created by the mitochondrial electron transport, as it leads to induction of genes associated with complexes I-IV of the electron transport chain [9]. To investigate this further, ATP levels were evaluated and phosphorylation of AMP kinase analyzed in T47D cells. Results indicate that UA treatment leads to decreased cellular levels of ATP after 24 hour treatment (5.0 µg/mL p = 0.0523, 10 µg/mL p = 0.010). As expected, the decreased levels of ATP were associated with increased phosphorylation of AMP kinase after both 24 and 48 hour treatment (10 µg/mL) (Fig. 1A and B).

This decline in cellular energy levels and triggering of the sensing mechanism would be expected to induce autophagy. Electron microscopy analysis of T47D cells treated with UA (2.5 and 5.0 µg/mL) for 24 hours indicated more marked presence of autophagic vacuoles, with double membranes characteristic of autophagosomes, compared with control (Fig. 2). These results were followed up by analysis for LC3 puncta by immunofluorescence, and an estimation of the abundance of autophagosomes, at different time points and treatments of three cell types (Fig. 3A, Fig. 3B and Fig. S2A–C). No effects were seen after treatment with UA (10 µg/mL) for 2 hours in any of the three cell lines, but an increase was observed after treatment with the anti-diabetic drug metformin in the T47D breast cancer cell line, which was no longer present following prolonged treatment. Metformin has previously been shown to stimulate AMP kinase already after one hour [28]. After 24 hours of treatment with UA (10 µg/mL) a significant increase in LC3 puncta was evident compared with controls in the two breast cancer cell lines. Immunoperoxidase staining of T47D cells also showed increased presence of LC3 puncta after UA treatment (Fig. S2D). These findings were further confirmed by observing an Increase in LC3 I and LC3 II by Western blotting (Fig. 3C). The effects on normal skin fibroblasts were not marked. For comparison, cells were starved by 40 min incubation in nutrient-free Hank’s balanced solution. Although visual inspection suggested presence of autophagosome formation in starved cells (Fig. 3A), LC3 puncta were difficult to count and this harsh treatment was not well tolerated by the cells.

Having observed an increase in LC3 puncta after treatment with UA, we investigated whether the formation of autophagic vacuoles was followed by autophagic flux. The levels of the autophagosomal cargo p62 were evaluated after exposure to UA. The concentration of p62 has been shown to diminish if autophagic flux is increased as it is degraded in the process [29]. Formation of autophagosomes as a result of treatment with UA after 24 hours (5.0 and 10 µg/mL) in T47D and MCF7 cells (Fig. 4A and Fig. S3) was not followed by degradation of internalized protein. The absence of p62 degradation at 24 hours suggests a disruption of lysosomal acidification and autophagolysosome maturation which could be caused by the proton shuttling properties of usnic acid across the lysosomal membrane, as seen in depolarization of the mitochondria [9], [10].

To evaluate further the effects of UA on lysosomes we used the lysosomal marker lysotracker in T47D cells which labels and tracks acidic organelles in cells. Results revealed very marked diffuse increase in lysotracker staining in T47D cells after UA treatment for 24 and 72 hours (Fig. 4B and Fig. 4C). This staining pattern has been interpreted as lysosomal dilatation as caused by treatment with chloroquine, which accumulates inside lysosomes. In cells treated with chloroquine, immunostaining for the lysosomal membrane protein Lamp1 copied the pattern obtained with lysotracker, thus confirming lysosomal dilatation [30]. In contrast, in our experiments, immunofluorescence staining for the lysosomal protein Lamp2 showed no morphological changes and no difference was observed between treated and untreated cells (Fig. 4D). This indicates that the lysotracker was staining outside the lysosome and could be explained by that fact that the retention of the dye inside of lysosomes depends on acidic pH [31]. The diffuse lysotracker staining following UA treatment might thus be due to protons being shuttled out of the lysosome, in a similar way as occurs across the mitochondrial membrane.

To explore autophagosome maturation following UA treatment, we utilized a plasmid construct, tfLC3 (mRFP-GFP-LC3 tandem-tagged fluorescent protein), with which we transfected the T47D cells. This method has previously been used to follow the autophagic maturation process. The GFP-LC3 loses fluorescence due to lysosomal acidity while the mRFP fluorescence is stable [23], [24]. Results showed that in starved cells GFP fluorescence was attenuated implying acidic conditions and degradation by lysosomal hydrolases, whereas mRFP fluorescence remained stable. After treatment with UA for 24 hours, GFP, as well as mRFP fluorescence was observed indicating disruption of autophagolysosomal acidification and impaired degradative conditions after treatment with UA (Fig. 4E). The failure of these cells to complete autophagy could contribute to the accumulation of autophagic vacuoles and retention of undegraded p62.

One of the adaptive features of most cancers is dysregulated pH. In normal cells intracellular pH is lower than the extracellular pH. In cancer cells the gradient is reversed creating a favorable environment for metastatic progression. Higher intracellular pH is maintained because of increased H^+^ efflux due to changes in the expression and/or activity of plasma membrane pumps and transporters [32]. The pH gradient in tumor cells is beneficial for the cellular accumulation of weak acids, such as usnic acid, causing weak acids to be mainly neutral at low pH and facilitating their transfer across the membrane. Treatment with UA shows significant induction of genes that are connected to complexes I through IV of the electron transport chain, which could be a compensating mechanism to preserve the proton gradient across the mitochondrial inner membrane [9], [32].

Studies of several inherited syndromes that predispose to various types of tumors and carcinomas have led to the identification of the mTOR pathway as a regulator of autophagy. Among downstream targets of mTORC1 are p70S6K and 4EBP1, which have an essential role in cell-cycle control and proliferation [33], [34]. In Fig. 1B we show that UA activates AMP-kinase, which signals to the mTORC1 complex. To explore downstream targets of mTORC1 we analyzed the effects in UA-treated cells on phosphorylated p70S6K by immunoperoxidase staining. The results showed a marked decrease in staining after treatment for 24 hours (Fig. 5A). Cells respond to nutrient shortage by inducing autophagy but this process can also be triggered by cellular stress [19]. To explore if UA could be triggering autophagy by other mechanism we tested for evidence of cellular stress in T47D cells after treatment with UA (10 µg/mL) for 6 hours. Increased phosphorylation of eIF2α, which is one of the recognized signs of ER stress, was detected (Fig. 5B).

The effects of usnic acid on autophagy can be compared with those described for the anti-malarial drug chloroquine which is currently in clinical trials in combination with anticancer regimens [19]. Chloroquine is a weak base (p*K*a 8.5) and accumulates inside lysosomes which become distended and dysfunctional, blocking autophagic flux [19]. In contrast, usnic acid is a weak acid that shuttles protons across membranes, thus increasing lysosomal pH, as shown by retention of the GFP signal, but lysosomal shape was not affected (LAMP2 staining). ER stress is induced by proteasome inhibition and ER-associated autophagy is therefore particularly relevant for cancer therapy with proteasome inhibitors. It has been shown that combining chloroquine with the proteasome inhibitor bortezomib increases tumor cell death *in vitro* and *in vivo* [35]. Cellular uptake and intracellular distribution of drugs is affected by pH [32], [36]. Chloroquine can e.g. prevent intracellular sequestration in lysosomes [36] but has no effect on mitochondrial accumulation of daunorubicin, suggesting that the compound does not affect mitochondrial pH [37]. As usnic acid affects pH in lysosomes and mitochondria it is predicted to influence intracellular drug distribution.

In conclusion, our previous study has shown that UA causes loss of mitochondrial membrane potential. In the current study, we have shown that this does not lead to release of cytochrome *c* and triggering of apoptosis. The H^+^ shuttling effect of UA operates at two organelles, mitochondria and lysosomes and its effect on autophagosome formation is likely to be triggered both by nutrition depletion and stress conditions. Autophagic flux is however incomplete and degradation of autophagosomal content does not occur. Our findings have implications for therapeutic manipulation of autophagy and pH-determined drug distribution.

## Supporting Information

Figure S1 **UA does not cause apoptosis.** (A) Cytochrome c leakage was not detectable, by immunofluorescense in T47D and Capan-2 cells after treatment with UA (10 µg/mL; DMSO 0.2%) for 24, 48 and 72 hours. (B) No cleavage products of Caspase-3 were detectable after treatment with UA (10 µg/mL; DMSO 0.2%) after 24, 48 and 72 hours. The scale bar shown represents 20 µm and applies to all panels.
(TIF)

Figure S2 **UA induces formation of autophagosome vacuoles.** LC3 puncta per cell were counted and quantified by ImageJ and data presented as 95% family-wise confidence level. (A) T47D cells treated with UA for 2 and 24 hours. (B) MCF7 cells treated with UA for 2 and 24 hours. (C) Normal human fibroblasts treated with UA for 2 and 24 hours. (D) An increase in LC3 immunoperoxidase staining was detected, in T47D cells after treatment with UA (10 µg/mL; DMSO 0.2%) for 24 hours. The scale bar shown represents 100 µm and applies to both panels.
(TIF)

Figure S3 **UA does not lead to degradation of p62.** No decrease in p62 immunoperoxidase staining was detected, in T47D cells after treatment with UA (10 µg/mL; DMSO 0.2%) for 24 hours. The scale bar shown represents 100 µm and applies to both panels.
(TIF)
