# Supplementary figures and images for: Proton-Shuttling Lichen Compound Usnic Acid Affects Mitochondrial and Lysosomal Function in Cancer Cells
Source: PLoS One. 2012 Dec 5;7(12):e51296. doi: 10.1371/journal.pone.0051296 (PMC3515546; doi:10.1371/journal.pone.0051296)

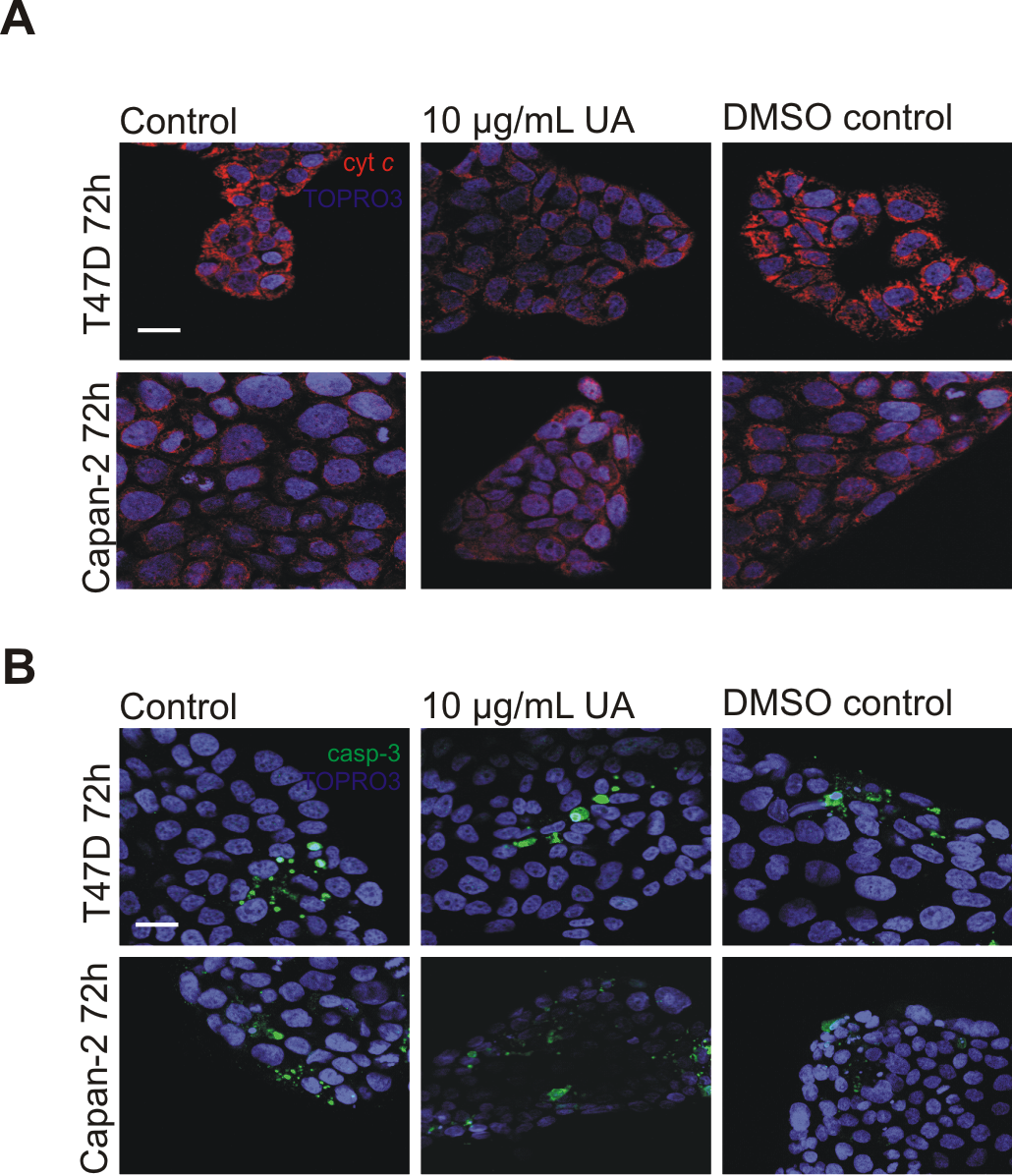

Supplement: Figure S1 — UA does not cause apoptosis. (A) Cytochrome c leakage was not detectable, by immunofluorescense in T47D and Capan-2 cells after treatment with UA (10 µg/mL; DMSO 0.2%) for 24, 48 and 72 hours. (B) No cleavage products of Caspase-3 were detectable after treatment with UA (10 µg/mL; DMSO 0.2%) after 24, 48 and 72 hours. The scale bar shown represents 20 µm and applies to all panels. (TIF) [file pone.0051296.s001.tif]

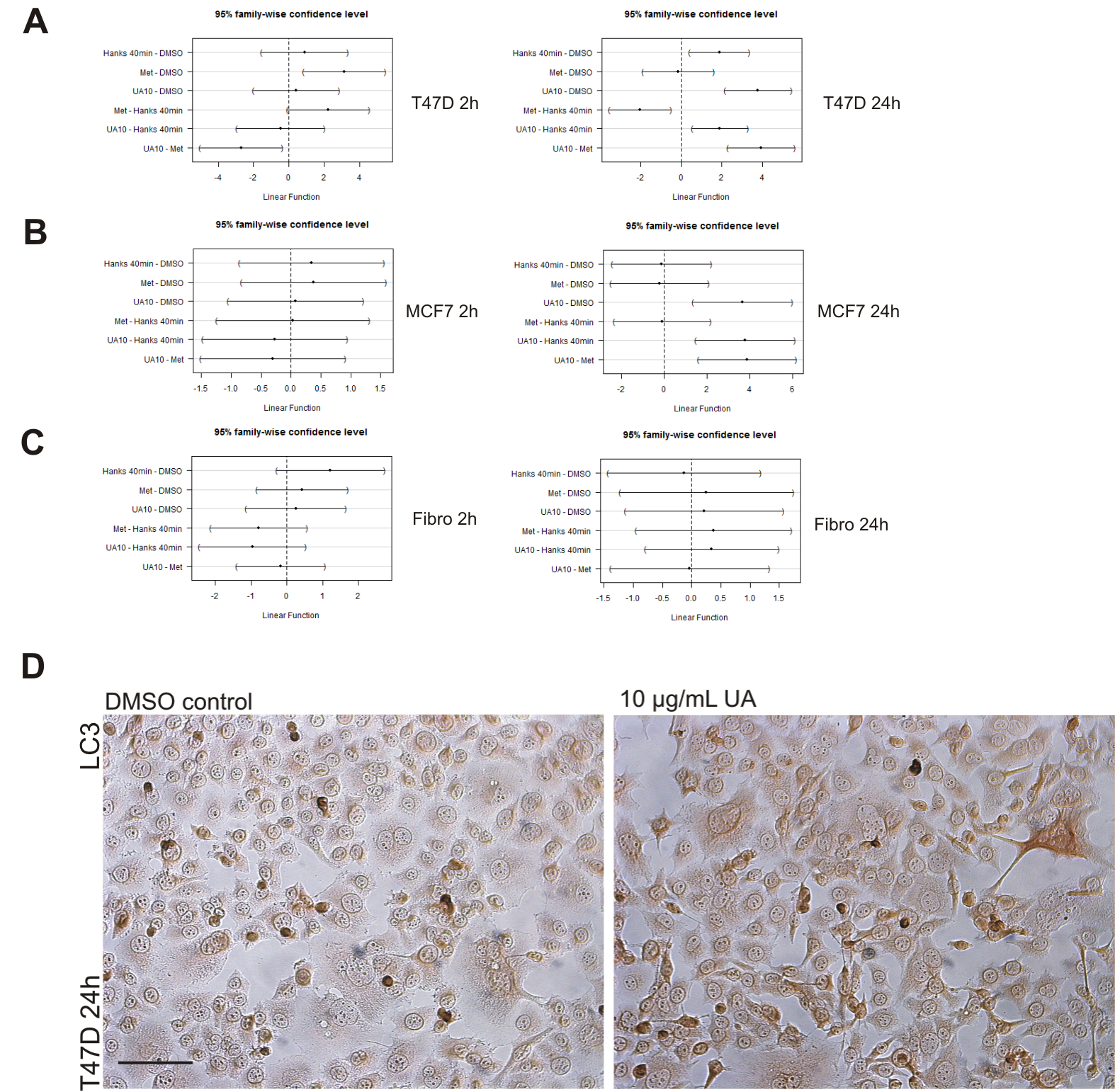

Supplement: Figure S2 — UA induces formation of autophagosome vacuoles. LC3 puncta per cell were counted and quantified by ImageJ and data presented as 95% family-wise confidence level. (A) T47D cells treated with UA for 2 and 24 hours. (B) MCF7 cells treated with UA for 2 and 24 hours. (C) Normal human fibroblasts treated with UA for 2 and 24 hours. (D) An increase in LC3 immunoperoxidase staining was detected, in T47D cells after treatment with UA (10 µg/mL; DMSO 0.2%) for 24 hours. The scale bar shown represents 100 µm and applies to both panels. (TIF) [file pone.0051296.s002.tif]

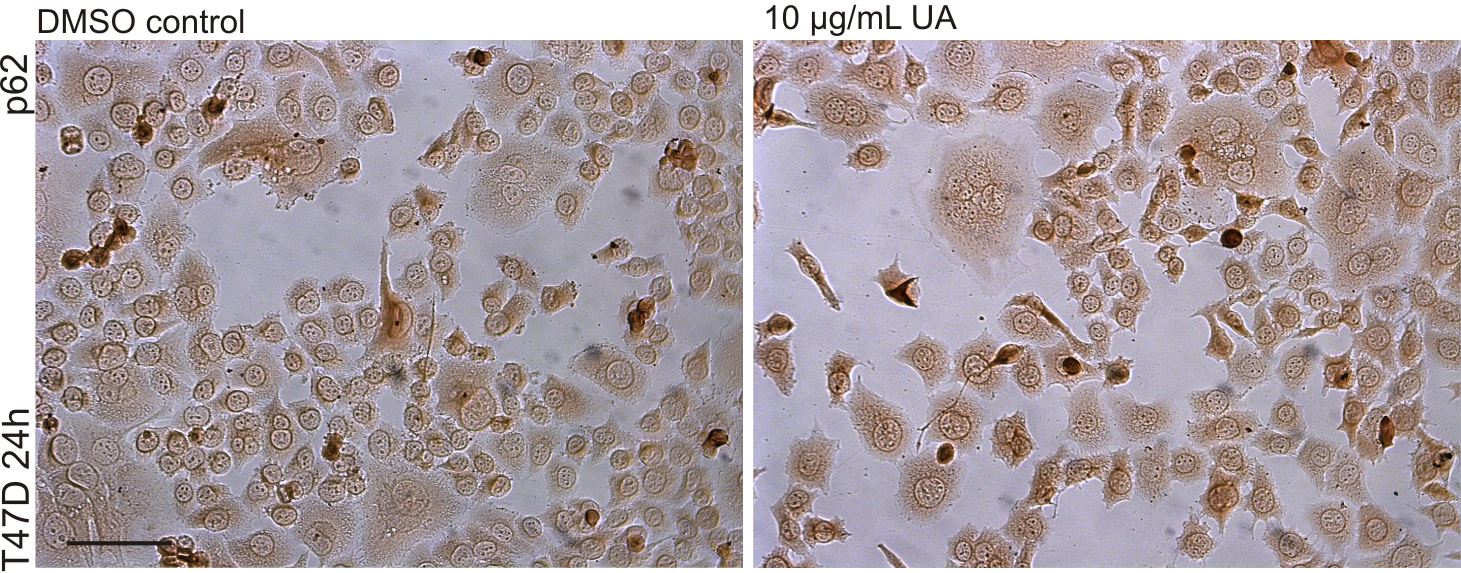

Supplement: Figure S3 — UA does not lead to degradation of p62. No decrease in p62 immunoperoxidase staining was detected, in T47D cells after treatment with UA (10 µg/mL; DMSO 0.2%) for 24 hours. The scale bar shown represents 100 µm and applies to both panels. (TIF) [file pone.0051296.s003.tif]
